# Transplantation of derivative retinal organoids from chemically induced pluripotent stem cells restored visual function

**DOI:** 10.1038/s41536-024-00387-7

**Published:** 2024-12-27

**Authors:** Ning Zhao, Chang-Jun Zhang, Xiao Zhang, Wen Wang, Kangxin Jin, Zi-Bing Jin

**Affiliations:** Beijing Institute of Ophthalmology, Beijing Tongren Eye Center, Beijing Tongren Hospital, Capital Medical University, 100730 Beijing, China

**Keywords:** Reprogramming, Induced pluripotent stem cells, Stem-cell differentiation

## Abstract

As an emerging type of pluripotent stem cells, chemically induced pluripotent stem cells (CiPSCs) avoid the risks of genomic disintegration by exogenous DNAs from viruses or plasmids, providing a safer stem cell source. To verify CiPSCs’ capacity to differentiate into retinal organoids (ROs), we induced CiPSCs from mouse embryonic fibroblasts by defined small-molecule compounds and successfully differentiated the CiPSCs into three-dimensional ROs, in which all major retinal cell types and retinal genes were in concordance with those in vivo. We transplanted retinal photoreceptors from ROs into the subretinal space of *retinal degeneration* mouse models and the cells could integrate into the host retina, establish synaptic connections, and significantly improve the visual functions of the murine models. This proof-of-concept study for the first time demonstrated that CiPSCs could differentiate into ROs with a full spectrum of retinal cell types, and provided new insights into chemical approach-based retinal regeneration for degenerative diseases.

## Introduction

Since the adult mammalian retina holds negligibly intrinsic regeneration capacities, retinal degenerative diseases, such as age-related macular degeneration (AMD), retinitis pigmentosa (RP), Leber congenital amaurosis (LCA), and Stargardt’s diseases, could lead to an irreversible loss of photoreceptor cells, thereby causing vision impairment and eventual blindness^1,2^. Currently, clinical interventions can temporarily slow down, but not reverse the deterioration of the diseases, and even worse is that there is no efficacious treatment for patients in the advanced stages^3^. The introduction of pluripotent stem cell-derived photoreceptors for cell replacement therapy provides a promising way to cure retinal degenerative diseases.

Photoreceptors play critical roles in the visual formation and physiology of the retina. Photoreceptors are the primary light-sensing cells and reside at the outer layer of the neural retina. The human retina contains one type of rod and three types of cones. The rods are responsible for dark vision, providing vision in low-intensity light conditions, whereas cones are responsible for bright vision, allowing trichromatic and high-acuity visual perception. Photoreceptors have obvious polarity with the outer segments at the apical side for photoelectric conversion and photoreceptor hyperpolarization. Then the hyperpolarization signal is transmitted through bipolar, and amacrine cells to retinal ganglion cells (RGCs), and the signal finally is transferred in the form of diverse parallel processing channels to the brain. As the central unit of visual perception, photoreceptors play an absolutely vital role in visual formation^1^.

Nowadays, various studies are focusing on obtaining donor photoreceptor cells for transplantation through three-dimensional (3D) retinal organoids (ROs)^2^. The RO is an organic conjugate of various cell types formed by the differentiation of pluripotent stem cells (PSCs) in a specific induction environment, exhibiting somewhat similar functionality as the retina in vivo. The ROs have shown great potential in cell transplantation studies. In 2011, Sasai team demonstrated the differentiation of first 3D ROs from mouse embryonic stem cells (ESCs) in vitro^4^. They generated fully stratified ROs which closely mimicked in vivo retinogenesis and consisted of all major neural retinal components including photoreceptors^4^, revealing a new approach for the production of photoreceptors in vitro. Subsequently, other groups transplanted mouse ESC- and mouse induced pluripotent stem cell (iPSC)-derived retinal cells into the subretinal space of advanced *retinal degeneration* mouse. The implanted graft survived well and formed direct synaptic connections with the host bipolar cells, which shed light on a more effective and lasting therapy strategy for advanced retinal degeneration^2^. Currently, several studies have been carried out to explore the application of human PSCs-derived 3D ROs in transplantation therapy^5,6^.

ESCs and iPSCs are frequently used in generating ROs, ensuring an almost infinite supply of cells for regenerative treatments. ESCs are established by expanding the inner cell mass cells isolated from blastocyst in vitro. However, in order to obtain ESCs, the embryo must be destroyed, which would lead to ethical problems. Moreover, ESCs are allogeneic sources which would trigger the acute and chronic immune rejection responses in host. Currently, a majority of iPSC lines are obtained through the ectopic expression of defined factors (Yamanaka factors). Although patient-specific iPSCs can avoid immune rejection and ethical problems, the genetic manipulation in reprogramming could potentially alter the genome which limits their clinical applications^7,8^.

Recently, Rouhani et al. found that strong selection pressure during somatic cell reprogramming to iPSCs by Yamanaka factors could introduce somatic mutations in iPSC lines, such as mutations in BCOR, a cancer-related gene^9^. This calls for more secure reprogramming methods such as using small chemical compounds which can redirect cell signaling pathways and thereby replace exogenous transcription factors to transform cell fates^10,11^. In 2013, Deng and colleagues successfully reprogrammed mouse embryonic fibroblasts (MEFs) into chemically induced pluripotent stem cells (CiPSCs) using a cocktail of seven small molecules without any exogenous genes. The CiPSCs resemble ESCs in terms of their gene expression profile, epigenetic status, and potential for differentiation and germline transmission^12^. Moreover, CiPSCs can be established from autologous somatic cells, and consequently no ethical issues or immune rejection problems will occur. Meanwhile, there are no exogenous transcription factors introduced during the reprogramming procedure. Therefore, it addresses the safety concerns that exogenous DNA fragments or viruses would disrupt genome integrity. Overall, this chemical reprogramming strategy is of great potential in generating functional cell types for clinical applications^12^.

However, CiPSCs were successfully established rather recently, and little is known about its in vitro differentiation capacity, specifically on 3D retinal differentiation. It is intriguing to identify the differentiation efficiency, the sensitivity to various small molecules, the expression of the related genes, and the functional integrity of tissues during CiPSCs differentiation. The CiPSC-derived 3D ROs constitute ideal cell sources for cell transplantation treatment, thereby providing a new strategy for retinal transplantation therapy for retinal degenerative diseases.

In this study, CiPSCs were established from MEFs by the chemical reprogramming method described previously^13^, and differentiated in 3D ROs^4,14–17^ with optimized protocols. It was observed that CiPSC-derived 3D ROs were polarized, stratified and contained all retinal cell types. The expression patterns of retinal genes were similar to those of in vivo. Importantly, the visual function of retinal degenerative mice was partially rescued by transplanted CiPSC-photoreceptors. This study established new approaches to induce somatic cells into retinal organoids by relying entirely on small molecules and thus provided new cell sources for clinical replacement therapy.

## Results

### Generation of CiPSCs by defined small-molecule compounds

**Fig. 1:**
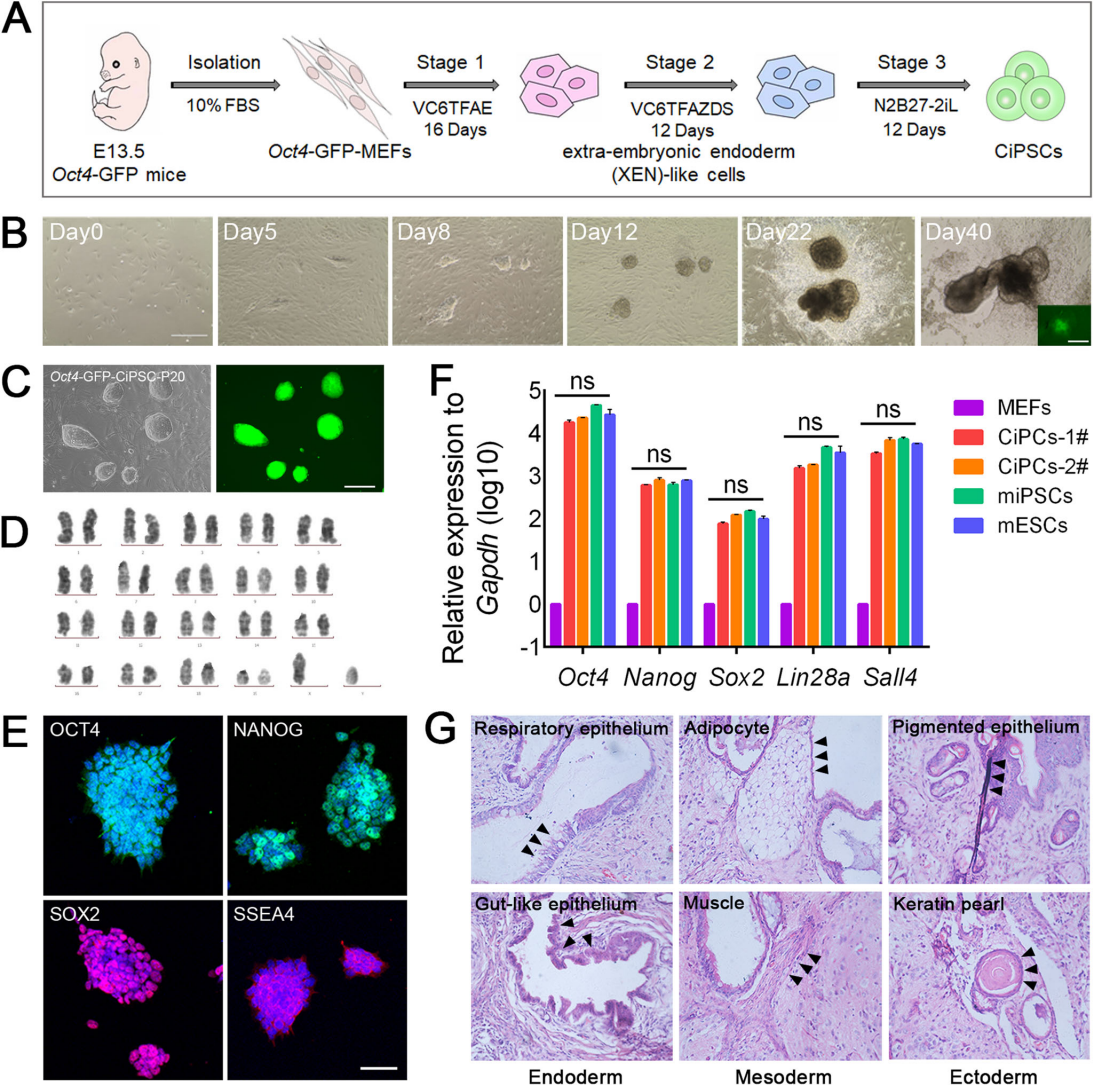
Generation of CiPSCs by defined small-molecule compounds. **A** Schematic diagram of the induction of *Oct4*-GFP-CiPSCs from MEFs. **B** Morphological changes during the induction of CiPSCs. The inset picture in D40 showed the expression of green fluorescence from *Oct4-GFP* cells. Scale bars, 400 μm. **C** Representative bright field and fluorescence images of the CiPSCs. Scale bar, 200 μm. **D** Karyotypic analysis of the CiPSCs colonies. **E** Immunostaining of pluripotency markers Oct4, Nanog, Sox2 and SSEA4 in CiPSC colonies. Scale bars, 50 μm. **F** The qRT-PCR analysis of mRNAs of pluripotency markers in MEFs, mESCs, miPSCs and two independent CiPSCs. Mean ± SEM, N = 3 independent experiments. **G** H&E staining of CiPSC-derived teratomas shows the presence of various structures from three germ layers. The black arrowhead pointed to the representative tissues of the three germ layers. See also Supplementary Fig. 1.

In order to effectively trace the generation of CiPSCs, *Oct4* promoter-driven green fluorescent protein (GFP) reporter MEFs (OG-MEFs), a reporting system for pluripotent marker, were employed. According to the previously developed method, it takes about 40 days to induce MEFs into CiPSCs by small-molecule compounds^13^ (Fig. 1A). As early as day 5 (D5, same abbreviation in below), there were a number of epithelial colonies formed. These colonies rapidly expanded and some of them expressed GFP at day 40, indicating that the cells have acquired pluripotency (Fig. 1B). At this point, these clones have not yet shown typical ESC-like morphology (domed, phase-bright, homogeneous with clear-cut edges) (Fig. 1B). Subsequently, these GFP^+^ colonies were picked up and seeded on feeder cells to establish CiPSC lines for further characterization and differentiation and defined as P1. These P1 clones maintained GFP expression and ESC-like morphology throughout passages (Fig. 1C, Supplementary Fig. 1A).

First, CiPSCs were identified to have normal karyotypes (Fig. 1D). Next, the pluripotent cell markers of CiPSCs were determined at mRNA and protein levels. The pluripotency-associated proteins Oct4, Nanog, Sox2, and SSEA4 could be identified by immunostaining of CiPSCs colonies (Fig. 1E). Meanwhile, the CiPSCs expressed pluripotency-associated transcripts such as *Oct4*, *Nanog*, *Sox2*, *Lin28a*, and *Sall4* and their expression levels were similar to those of mESCs (Fig. 1F). Last, the differentiation potentials of CiPSCs were further examined. In vitro differentiation test showed that CiPSCs could differentiate into different cell types of three germ layers (Supplementary Fig. 1B). As previously described, teratoma formation experiment is the gold standard for verifying whether the cells possess pluripotency, and HE staining is a commonly used method for identifying the germ layers of teratoma^12,13^. CiPSCs were able to form teratoma after injected into immune-deficient SCID mice, and we could identify representative tissues of the endoderm, mesoderm, and ectoderm in the generated teratoma by HE staining, such as the endoderm: pseudostratified ciliated columnar epithelioid respiratory epithelium, and stratified squamous epithelioid gastrointestinal epithelium, the mesoderm: the large, round and empty adipose tissue, and cylindrical, multinucleated, striated muscle tissue, the ectoderm: blackened melanin tissue, and rich cytoplasm, acidophilic keratinized beads, which confirmed that the injected cells have the ability to differentiate into three germ layers, demonstrating their pluripotency (Fig. 1G). These findings demonstrated that CiPSCs were fully pluripotent as other typical iPSC lines.

### Generation of polarized retinal organoids from CiPSCs

**Fig. 2:**
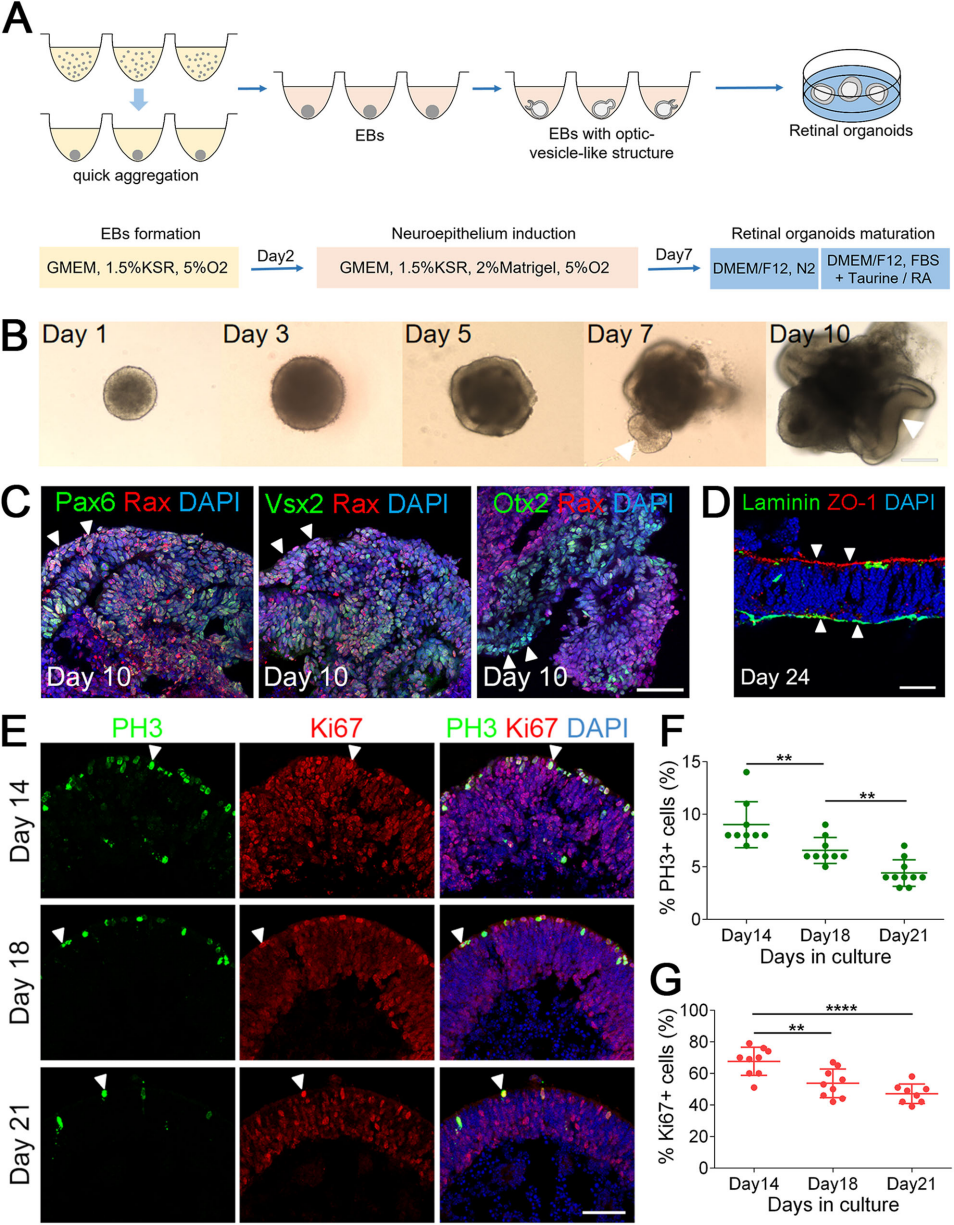
Generation of polarized neuroretinal tissues from CiPSCs. **A** Schematic diagram of the methods for ROs differentiation from CiPSCs. **B** Morphological changes in early differentiation stages of ROs from CiPSCs. Arrowheads point to the optic vesicle and/or optic cup structures. Scale bars, 200 μm. **C** Retinal progenitor cells were shown by immunostaining with Rax (red), Pax6 (green), Vsx2 (green) and Otx2 (green). RPE progenitors were shown by Otx2^+^; Rax^-^ immunostaining. Arrowheads indicate relevant colocalized cells. Scale bar, 50 μm. **D** Immunostaining of ROs with the apical (ZO-1) and basal (Laminin) markers on D24. Arrowheads show the apical side (ZO-1^+^) and the basal side (Laminin^+^) of organoids. Scale bar, 25 μm. **E** A gradual decline of cell proliferation at distinct timepoints during differentiation. Arrowheads indicate relevant PH3^+^ (green) and Ki67^+^ (red) cells. Images were captured using the same confocal settings. Scale bar, 50 μm. **F**, **G** Quantitative analysis of PH3^+^ and Ki67^+^ cells. Data was represented as means ± SEM. n > 8, **p < 0.01, ****p < 0.0001. See also Supplementary Fig. 1.

We optimized previously reported methods to differentiate 3D ROs (Fig. 2A)^4,18^. During the formation of embryoid bodies (EBs), the majority of cell aggregates developed neuroepithelium at D5, and a few optic vesicle-like structures appeared at D7 (Fig. 2B). For ROs differentiation, the D7 EBs were transferred into retinal maturation medium, and more optic cup-like structures formed at D10 (Fig. 2B). The optic cups contained retinal progenitors (Rax^+^, Pax6^+^, Vsx2^+^) and retinal pigment epithelium (RPE)progenitors (Otx2^+^, Rax^−^) (Fig. 2C). Furthermore, the neural retinae were polarized with the inner side to be basal (Laminin^+^) and the outer side apical (ZO-1^+^) (Fig. 2D). When tracing the differentiation of EBs from *Oct4*-GFP^+^ CiPSCs, we observed that the GFP^+^ signal gradually decayed and completely disappeared at D8 (Supplementary Fig. 1C).

To further examine the cell proliferation during differentiation, we performed the immunostaining of organoids for PH3 and Ki67 expression (Fig. 2E). Both PH3^+^ and Ki67^+^ cells were gradually decreased from D14 to D21. The proportion of positive cells at D14, 18 and 21 is 9.00 ± 0.73%, 6.56 ± 0.41% and 4.40 ± 0.40% for PH3, and 67.67 ± 3.00%, 53.67 ± 3.01% and 47.13 ± 2.18% for Ki67 (means ± SEM. N > 8), respectively (Fig. 2F, G). Taken together, these results demonstrated that the cells in organoids gradually exited the cell cycles and differentiated into various retinal cell types.

### Morphological and molecular characterization of CiPSC-derived retinal organoids

**Fig. 3:**
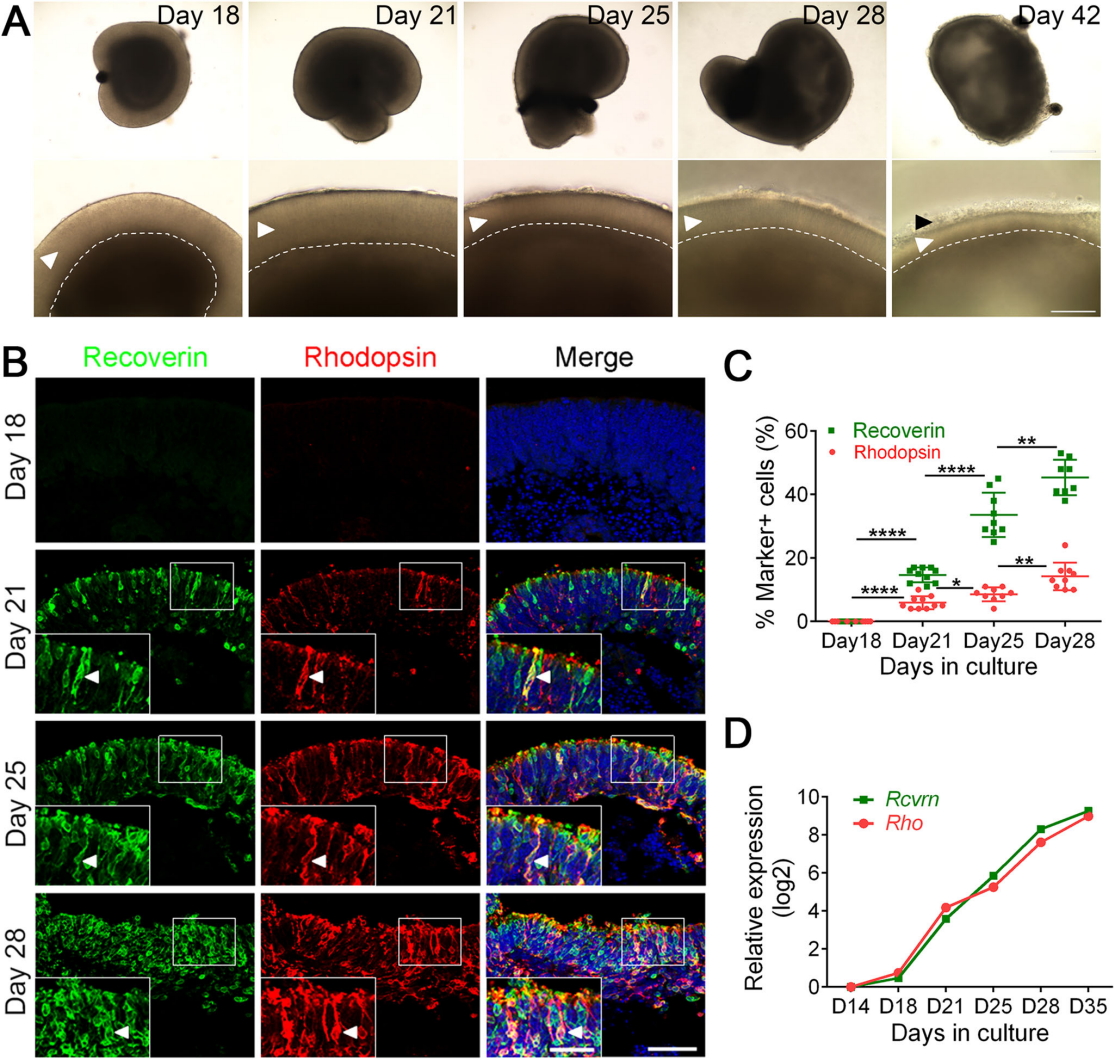
Morphological and molecular characterizations of CiPSC-derived retinal organoids and RPE. **A** Morphological changes of ROs at different stages of differentiation. The amplified neural retina of the corresponding days was displayed in the bottom panel. The curves indicate the boundaries of photoreceptors. The polarized neuroretina displayed significant layered structure and the thickness of the photoreceptor became thinner with time. The white arrowhead pointed to the neuroretina structures. The black arrowhead pointed to the outer segment (OS)-like structures. Scale bar, 400 μm (top), 100 μm (bottom). **B** Immunostaining of Recoverin (photoreceptor precursor cell marker, in green) and Rhodopsin (rod photoreceptor cell marker, in red) in various stages of ROs. Indicated rectangle regions were amplified as inset pictures in the bottom left. Arrowheads indicate Recoverin and Rhodopsin double-positive cells which represent rod photoreceptors. Scale bars, 25 μm (left, for insets), 50 μm (right). Images were captured using the same confocal settings. **C** Quantitative analysis of Recoverin^+^ and Rhodopsin^+^ cells. Data was represented as means ± SEM. N ≥ 8, *p < 0.05, **p < 0.01, ****p < 0.0001. **D** The qRT-PCR analysis of the dynamic expression of photoreceptor cell-related genes *Rcvrn* and *Rho* during organoid differentiation. The average expression value for each gene was further normalized to that of D14 to yield relative expression values (log2 scale). Data was represented as means ± SEM. See also Supplementary Fig. 2.

Consistent with previously reported results^4,19^, in this research, retinal organoids gradually formed big epithelial structures with clear stratification during differentiation, and the thickness of the photoreceptor layer became thinner with time (Fig. 3A). At the late stage of differentiation, outer segment (OS)-like structures were observed at the outer surface of organoids (Fig. 3A), which corresponded to the photoreceptor outer segments in vivo^20^.

To investigate the morphology of photoreceptors, we immunostained ROs for photoreceptor markers Recoverin and Rhodopsin. It showed that Recoverin^+^ and Rhodopsin^+^ cells were detectable at D21, and both Recoverin^+^ and Rhodopsin^+^ cells increased as differentiation proceeded (Fig. 3B). Interestingly, Recoverin and Rhodopsin were located at the apical side of the neural retinal (Fig. 3B), consistent with photoreceptor morphogenesis in vivo. The proportion of positive cells at D18, 21, 25 and 28 was 0.0 ± 0.0%, 14.70 ± 0.73%, 33.56 ± 2.33% and 45.38 ± 1.98% for Recoverin, and 0.0 ± 0.0%, 5.90 ± 0.64%, 8.56 ± 0.73% and 14.22 ± 1.45% (means ± SEM. N > 8) for Rhodopsin, respectively (Fig. 3C), indicating the gradual maturation of the photoreceptors during differentiation. At the transcriptomic level, qRT-PCR analysis of photoreceptor cell-related genes *Rcvrn*, *Rho* and *Nrl* showed a continuous increase of their expression during differentiation (Fig. 3D, Supplementary Fig. 2A, B), which was also in concordance with the developing mouse retina in vivo (Supplementary Fig. 2A, B).

Overall, these findings suggested that CiPSCs could differentiate into retinal organoids with polarized photoreceptor layers, which highly resembled in vivo photoreceptor genesis.

### Development and maturation of retinal organoids

**Fig. 4:**
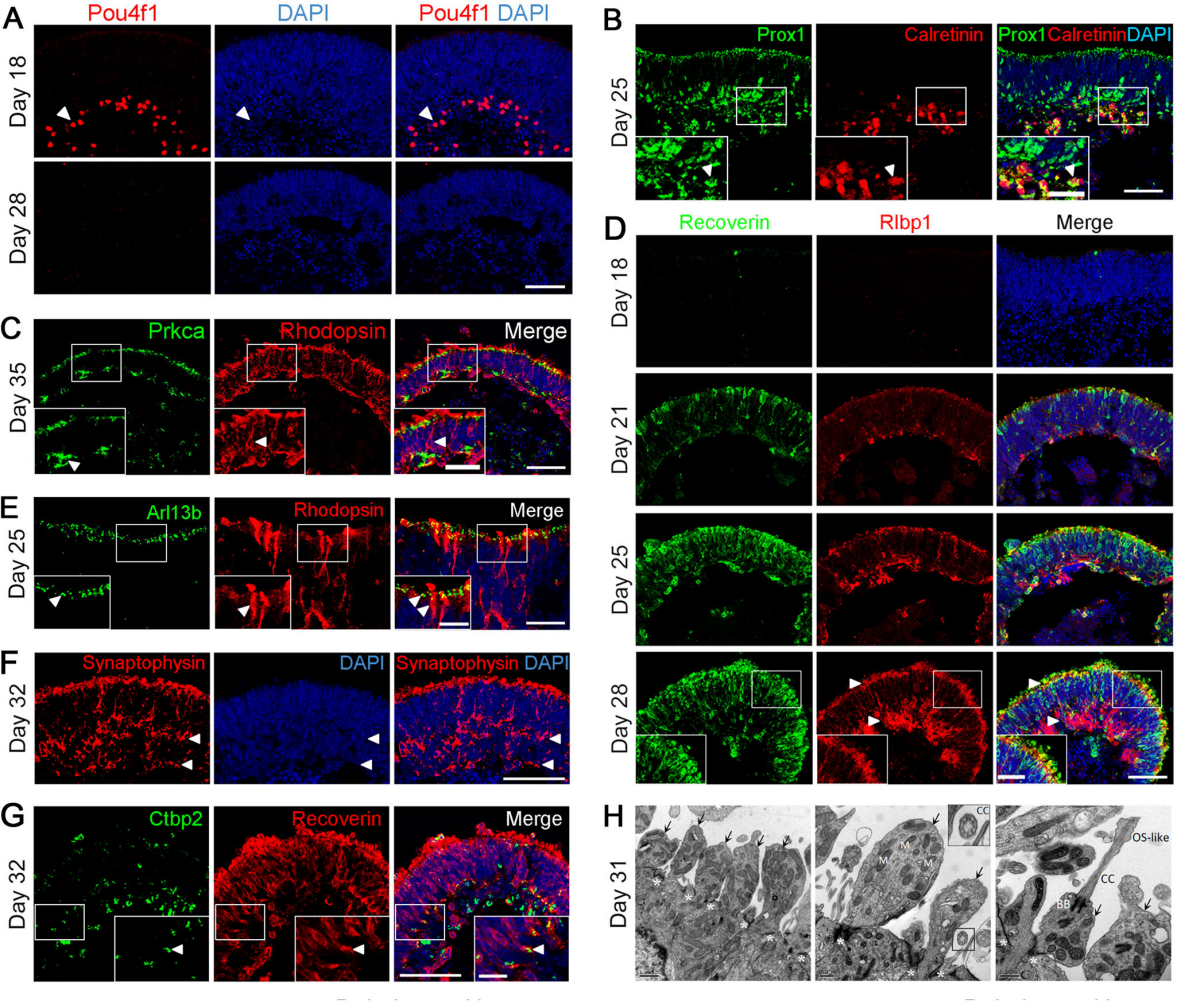
Development and maturation of retinal organoids. **A** Retinal ganglion cells (RGCs) indicated by Pou4f1 (red) at D18 and D28 in ROs. Arrowheads indicate relevant immunostaining with Pou4f1. Scale bar, 50 μm. Images were captured using the same confocal settings. **B** Immunostaining of ROs for Prox1 (horizontal/amacrine cell marker, in green) and Calretinin (amacrine/ganglion cell marker, in red). Arrowheads indicate Prox1^+^, Calretinin^+^ double-positive cells representing amacrine cells. Scale bars, 25 μm (left, for insets), 50 μm (right). **C** Immunostaining of ROs for Prkca (rod bipolar cell marker, in green) and Rhodopsin (rod photoreceptor cell marker, in red). Arrowheads indicate relevant immunostaining with Prkca and Rhodopsin. Scale bars, 25 μm (left, for insets), 50 μm (right). **D** Müller glial cells indicated by Rlbp1 (red) in the ROs. Recoverin (green) is a marker for photoreceptor precursors. Arrowheads indicate relevant immunostaining with Rlbp1. Scale bar, 50 μm. Images were captured using the same confocal settings. **E** Immunostaining of organoids with connecting cilium marker Arl13b (green) and rod photoreceptor marker Rhodopsin (red). Arrowheads indicate relevant immunostaining with Arl13b and Rhodopsin. Scale bars, 5μm (left, for insets), 10 μm (right). **F** Immunostaining of organoids with synaptic vesicle marker Synaptophysin (red). Arrowheads indicate relevant immunostaining with Synaptophysin. Scale bars, 50 μm. **G** Ctbp2 (green) and Recoverin (red) are markers for synapse and photoreceptor precursors, respectively. Arrowheads indicate relevant immunostaining with Ctbp2 and Recoverin. Scale bars, 50 μm (left), 12.5 μm (right, for insets). **H** Transmission electron microscopy (TEM) imaging of ROs showed outer limiting membrane (*), inner segments (arrows) mitochondria (M), connecting cilia (CC), basal bodies (BB), and OS-like structures. Scale bars, 1 μm (left), 0.5 μm (middle, right). See also Supplementary Fig. 2.

Retinal neurons form stratified structures called inner and outer plexiform layers which are essential for visual signal processing and transferring. On D18, Pou4f1, a marker for RGCs, was detected at the basal side of organoids (Fig. 4A). However, RGCs could not survive throughout the later stages of differentiation (Fig. 4A), probably due to the lack of axon formation^21^ and/or the insufficiency of oxygen and nutrients^22^ during differentiation, which also happened in other 3D ROs differentiation systems^19^.

Prox1 (a marker for horizontal and amacrine cells) and Calretinin (a marker for amacrine and ganglion cells) were detected on D25. Both signals were polarized at the basal side of organoids, and the Calretinin^+^ cells were located at the inner side of Prox1^+^ cells, which was the same as the distribution of horizontal cells, amacrine cells, and RGCs in the mouse retina (Fig. 4B). At the same time, the Prox1^+^, Calretinin^+^ double-positive cells indicated the generation of amacrine cells (Fig. 4B). As the bipolar cells are late-born cell type during mouse retinal development^20^, Prkca, a marker of rod bipolar cells, can only be detected at the late stage of differentiation (Fig. 4C). Moreover, most Prkca^+^ signals were located at the basal side of the neural retina and the inner side of Rhodopsin^+^ cells, reflecting the correct distribution of bipolar cells and rod photoreceptors in ROs (Fig. 4C).

Müller glia cells also have essential tasks in maintaining retinal homeostasis and photoreceptor function. On D21, Rlbp1, a marker for Müller glia cells, was detected at the basal side of organoids. The positive signals spanned the whole neural retina and formed the outer limiting membrane (OLM) at the apical side by D28 (Fig. 4D), which was similar to in vivo retinogenesis. Rhodopsin^+^ rod photoreceptors grew beyond the OLM (Fig. 4D), indicating the formation of the cilia and outer segments.

To examine the maturation of photoreceptors, we conducted immunostaining of organoids for cilium and synapse-related proteins. The connecting cilia marker Arl13b was detected at the apical side of the neural retina, co-labeled with Rhodopsin^+^ rod photoreceptors (Fig. 4E). Meanwhile, some Rhodopsin^+^ signals grew beyond the Arl13b^+^ cilia (Fig. 4E), indicating the formation of the outer segments. Synaptophysin is a marker for synaptic vesicles, normally labeling the outer plexiform layer (OPL) and inner plexiform layer (IPL) in the neural retina. The immunostaining result showed a stratified distribution of synaptophysin staining, representing the OPL and IPL (Fig. 4F) and reflecting the maturation of photoreceptors. In addition, Ctbp2 is a presynaptic protein that typically stains at the rod terminal in the ribbon synapse^23^. Ctbp2 staining was detected at the basal side of the neural retina on D32 and co-localization of Ctbp2 with Recoverin indicated the formation of synaptic connections between photoreceptors and bipolar cell types (Fig. 4G).

The ultrastructures of photoreceptors were further characterized by transmission electron microscopy (TEM)^24^. Numerous components of mature photoreceptors such as OLM, connecting cilia (CC), basal cilia (BC), mitochondria-rich inner segment (IS), and outer segment (OS)-like structure could be identified in the TEM images (Fig. 4H).

At the mRNA level, the qRT-PCR analysis of RGCs (*Pou4f2*, *Atoh7*), interneurons (*Neurod1*), and Müller glia (*Rlbp1*) related genes showed an increase of gene expression during differentiation, which conformed to the developmental mouse retina in vivo (Supplementary Fig. 2C–E).

These results suggested that CiPSC-derived 3D ROs contained all types of examined retina cell types, including photoreceptors, RGCs, horizontal, amacrine, bipolar and Müller glial cells, and the genesis and distribution of different cell types recapitulated the mouse retinogenesis in vivo. More importantly, the CiPSC-derived photoreceptors should have the ability to receive and transfer visual information by the successful formation of intact structures.

### Dynamic transcriptomic profiling of retinal organoids

**Fig. 5:**
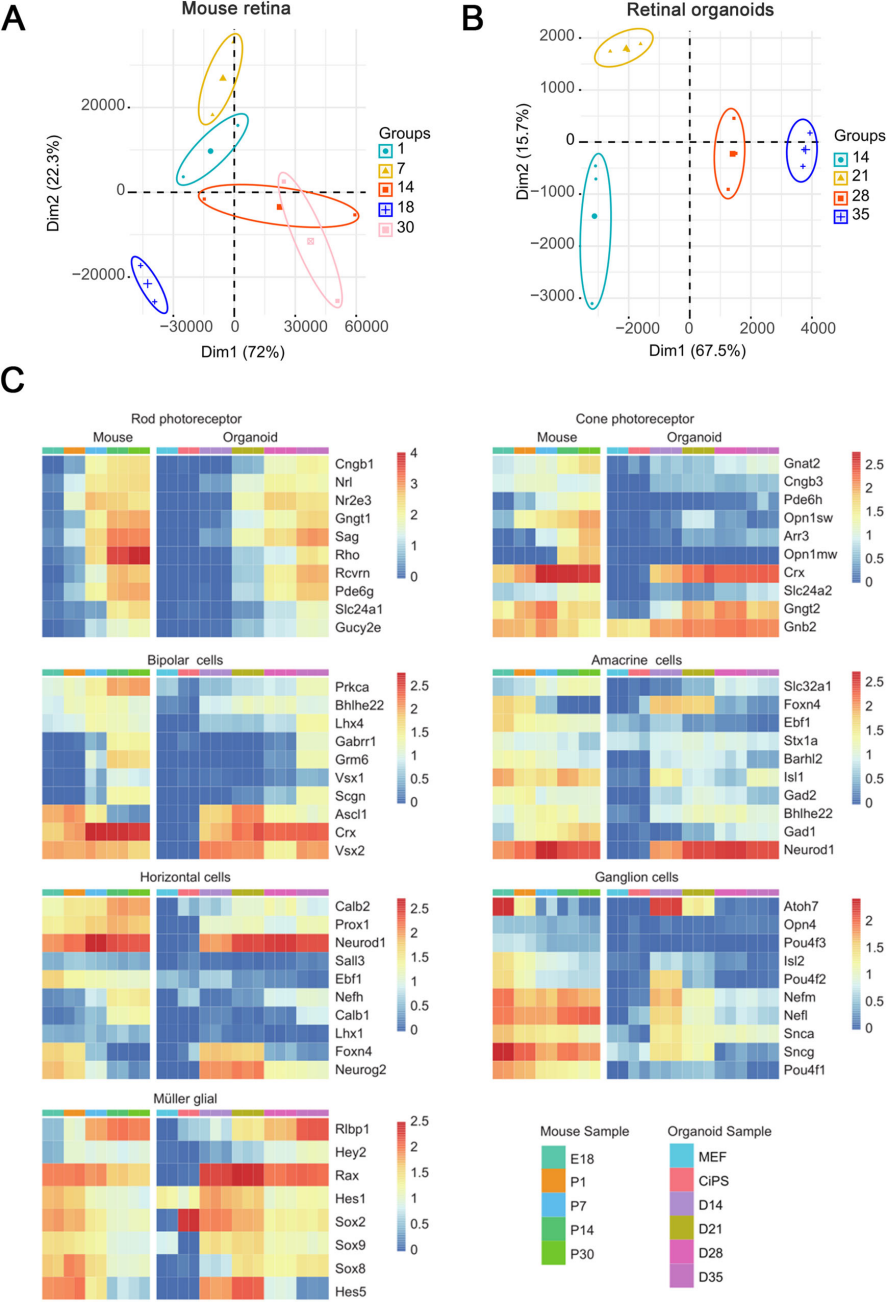
Dynamic transcriptome analysis of developing retinal organoids. **A**, **B** The principal component analysis (PCA) of the expressed CPM values for samples from day 14, 21, 28, and 32 of retinal organoids and embryonic day 18 (E18), postnatal day 1(P1), P7, P14, and P30 in vivo mouse retinae. Percentages indicate experimental variance assigned to each PC. **C** Heatmaps showing the expression of selected cell type-specific genes in retinal organoids and in vivo developing mouse retinae. The average expression value (counts per million [cpm]) at each timepoint is plotted in log2 scale. The color scale bars refer to gene expression. Three biological sample replicates were used at each timepoint. See also Supplementary Fig. 3.

To characterize the transcriptome during retinal development, RNA-seq of CiPSC-derived ROs (D0, 14, 21, 28, and 32) and in vivo mouse retinae (embryonic day 18 (E18), postnatal day 1(P1), P7, P14, and P30) was performed (Fig. 5A–C and Supplementary Fig. 3). The principal component analysis (PCA) of these samples indicated that developmental timing is the most important factor, which, as the first principal component (PC1), can explain 72% and 67.5% of the variance in the two datasets (Fig. 5A, B).

The expression of selected cell-type-specific genes in ROs and in vivo mouse retinae was further analyzed (Fig. 5C, Supplementary Fig. 3). Based on IHC data, gene expression patterns during organoid differentiation showed a remarkable resemblance to those of the developing mouse retina in vivo (Fig. 5C, Supplementary Fig. 3A). Rod and cone photoreceptor genes showed a progressive increase with differentiation progress, which was consistent with in vivo retinogenesis. Changes in horizontal, amacrine, bipolar, and Müller glial cell-specific gene expression in organoids revealed a striking coincidence with in vivo retinae, suggesting that the CiPSC-derived ROs closely recapitulated in vivo development. Moreover, expression of RGC-specific genes was downregulated with differentiation and was hardly detectable in organoids after D21 (Fig. 5C, Supplementary Fig. 3A), consistent with their disappearance in organoids. Moreover, Pearson correlation analysis between retinal organoids and mouse retinae showed that the transcriptomes of D21 ROs correlated strongly with P1 and P7 mouse retinae (Supplementary Fig. 3B). Likewise, correlation was high among transcriptomes between D14 ROs and E18 mouse retinae, D28 ROs and P7 mouse retinae, D35 ROs and P14 mouse retinae, respectively (Supplementary Fig. 3B). These results suggested that the gene expression patterns of organoids were in concordance with the developing mouse retina in vivo.

### Generation of *Crx*-tdTomato labeled photoreceptors from CiPSCs

**Fig. 6:**
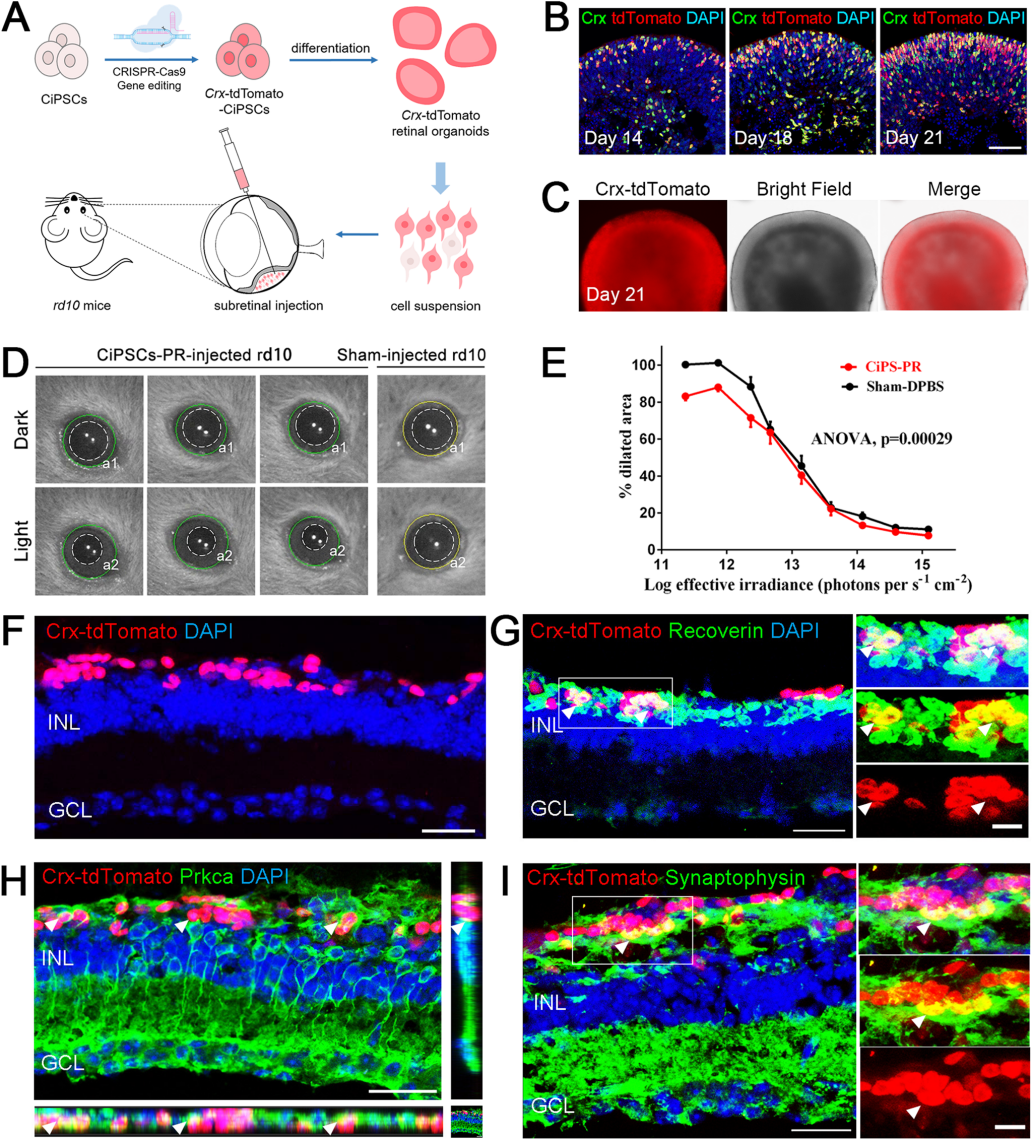
Transplantation of CiPSC-derived *Crx*-tdTomato^+^ photoreceptors into *rd10* mice. **A** Schematic of the generation of *Crx*-tdTomato reporter CiPSCs and the transplantation of CiPSC-derived photoreceptors into *rd10* mice. **B** Immunostaining of photoreceptor marker Crx in *Crx*-tdTomato ROs. Images were captured using the same confocal settings. Scale bar, 50 μm. **C** Representative bright field and fluorescence images of the *Crx*-tdTomato reporter CiPSC-ROs. Scale bar, 200 μm. **D** Representative infrared images of pupil areas measured in dark (a1) and light (a2) corresponding to dashed-line circles in 30 days of post-transplantation *rd10* mice that received CiPSC-PR in one eye and a sham injection (DPBS) in the other at P24. **E** Pupillary response plots (a2/a1) against the different intensities of irradiance for **D**. N = 9 for both groups, ANOVA, mean ± SEM. **F** Integration and survival of *Crx*-tdTomato^+^ photoreceptors in the *rd10* mouse retina at 2 months post-transplantation. Scale bars, 25 μm. **G** Orthogonal view of Recoverin (green) and *Crx*-tdTomato (red) double-positive cells (arrowhead). Scale bars, 25 μm (left), 10 μm (right). **H** Representative image to show that host bipolar cells (Prkca^+^, green, arrowhead) connected with *Crx*-tdTomato (red) photoreceptors at the tip of dendritic processes. Scale bars, 30 μm. **I** Representative image to display presynaptic formation at the host-graft interfaces (arrowheads). Scale bars, 25 μm (left), 10 μm (right). See also Supplementary Fig. 4.

The *Crx* plays an important role in specifying the fates of photoreceptor cells. *Crx* expression is first detected in photoreceptor precursors and then continues to express in all photoreceptors as the differentiation proceeds^25^. In order to track the development of photoreceptor cells, we constructed a *Crx*-reporter CiPSC line by inserting the coding sequence of the tdTomato fluorescent protein prior to the stop codon of *Crx* gene with CRISPR/Cas9 technology (Fig. 6A, Supplementary Fig. 4A–C).

From D14 onwards, both the number of tdTomato^+^ photoreceptors (Fig. 6B, Supplementary Fig. 4D) and the tdTomato intensity (Supplementary Fig. 4E) kept increased, especially at the apical side. The tdTomato^+^ signals colocalized with Crx^+^ cells by immunostaining (Fig. 6B), indicating that insertion of tdTomato coding sequence did not alter the expression pattern of *Crx* gene. Meanwhile, the percentage of tdTomato^+^ cells constituted around 85% of all cells in the ROs at D32 (Supplementary Fig. 4E), which was consistent with the mature mouse retina in vivo^20^. Additionally, qRT-PCR analysis of *Crx* also showed a continuous increase of *Crx* expression during differentiation, which was also in concordance with the developmental mouse retina in vivo (Supplementary Fig. 4F). Furthermore, almost all organoids strongly expressed *Crx*-tdTomato on D21, demonstrating the consistency in generating tdTomato^+^ photoreceptors (Fig. 6C). Therefore, the photoreceptors in retinal organoids could be reflected and recorded by tdTomato reporter during differentiation.

### Transplantation of CiPSC-derived photoreceptors into *rd* mouse model

To determine whether CiPSC-derived photoreceptors (CiPSC-PR) can restore visual function, single *Crx*-tdTomato^+^ photoreceptor cell suspension was transplanted into the subretinal space of P24 *rd10* mice, a mouse model of retinal degeneration (Fig. 6A). According to previous studies of our lab, cell sorting operations would lead to severe decline of cell states and affecting the survival and integration of transplanted cells in the host retina. Therefore, in this experiment, digested single cells were directly transplanted without sorting to ensure better cell survival and integration. For comparison, the P24 *rd10* mice received *Crx*-tdTomato^+^ donor cells in one eye and sham injections (DPBS) in the other (N = 9). Four weeks after transplantation, the pupillary light reflex (PLR) test was conducted to measure photoreceptor function because the light-induced pupil constriction is a behavioral response that depends on photoreceptors’ functional connections with central brainstem targets^26,27^ (Fig. 6D). The results showed that the eyes received *Crx*-tdTomato^+^ cells had a more sensitive pupil reflex compared with the sham-injected eyes, especially at low light intensities (Fig. 6E).

Three months after CiPSC-PR transplantation, the eyes were examined by immunofluorescence for evidence of functional integration into the host retina. The tdTomato^+^ or tdTomato^+^ Recoverin^+^ cells were distributed in the ONL (Fig. 6F, G). The Prkca^+^ rod bipolar cells were in close proximity to tdTomato^+^ cells, indicating the potential formation of synaptic connections between graft photoreceptor cells and host rod bipolar cells (Fig. 6H). Moreover, CiPSC-PR were found to form synaptic terminals that express the synaptic vesicle protein synaptophysin, and synaptophysin^+^ synapses were consistently located in the area of the host-graft interface as well as the IPL within the host retina (Fig. 6I), which is essential for transmitting the light signals into the inner retina.

These results demonstrated that some of the transplanted CiPSC-PR integrated into the host retina, formed functional synaptic connections with downstream retinal neurons, and contributed to visual function.

## Discussion

This study reported small-molecule compounds reprogramming approaches that could convert fibroblasts to 3D ROs with mature neurons, mimicking in vivo retinogenesis. The obtained CiPSCs resembled ESCs in terms of their morphology, proliferation capacity, gene expression profile, and differentiation potential. In addition, CiPSCs could differentiate into polarized and stratified 3D ROs that contained all major retinal cell types, including photoreceptors, RGCs, horizontal, amacrine, bipolar and Müller glial cells. The presence of cilia and outer segments also verified the good maturation status of photoreceptors. The expression patterns of retinal genes revealed that the CiPSC-ROs highly resembled in vivo retinogenesis. More importantly, CiPSC-derived photoreceptors were able to restore light sensitivity in the degenerative retinae.

The emergence of iPSC technology brings unprecedented development for cell transplantation therapies. Compared with ESCs, iPSCs not only bypass ethical issues, but also circumvent graft-related immune rejections, providing individualized treatment options. Traditional induction of iPSCs depends on the introduction of exogenous genes, i.e., OSKM by retroviruses or lentiviruses^7,8^. Although the four transcription factors were almost silenced in the end, *c-Myc* retrovirus reactivation was detected in the tumors in a few cases^28^. Moreover, these transcription factors are integrated by viruses into the ‘random’ genomic sequences of target cells, resulting in possible risks of gene activation, inactivation, or insertional mutations. The integration-free methods, e.g., via episome or Sendai virus, theoretically reduce but still cannot completely rule out the possibility of exogenous DNA fragments left in the genome^29,30^. Other options include the induction by mRNAs and/or the recombinant proteins^31^. Besides the cost and efficiency issues, these protocols generate inconsistent batch-to-batch results due to the instability nature of mRNAs and proteins in vitro.

Small-molecule compounds possess many advantages in cell reprogramming. They are non-immunogenic, more cost-effective, more precise regulation, and easier to synthesize, store and standardize. Most importantly, through chemically remodeling epigenetic modifications and regulating specific signaling pathways, small-molecule compounds reprogramming thoroughly eliminates the risks of genome disruption associated with genetic manipulation^10,12,32^. After the first CiPS cell line via chemical approach was established^12^, various subsequent studies have confirmed the feasibility of this chemically reprogramming^33,34^. In 2015, Deng et al. further improved the induction efficiency by manipulating small molecules more precisely^13^. Therefore, we adopted this optimized strategy to generate CiPSCs.

In this study, the CiPSC-ROs contained almost all neural retinal cell types, including photoreceptors, RGCs, horizontal, amacrine, bipolar and Müller glial cells. Meanwhile, RGCs could not be detected at the later stages of differentiation in CiPSC-ROs, which was consistent with the findings of other studies^19,35^. As RGCs possess axonal connection toward the optic nerve in the in vivo retina, but there is no optic nerve generation in ROs, the maintenance of RGCs could be affected by the lack of axon formation in ROs^21^. At the same time, RGCs are distributed in the inner side of ROs, since the supply of oxygen and nutrients in the inner part of the organoids is significantly lower than that in the outer side, which also affects the survival of RGCs^22,36^. Recently, a study reported that the transport of oxygen and nutrients could be improved by bioreactors^19^. Future studies could focus on the coculture of 3D ROs with different tissues (e.g., brain organoids, vascular tissues) to promote the survival of RGCs^37^.

Moreover, the ultrastructures of photoreceptors demonstrated the formation of cilia and nascent outer segments (OS) in CiPSC-ROs. Although many OS-like structures could be seen at the outer surface of organoids under the bright-field microscope, there were rare typical membrane discs of the outer segments observed by TEM. The main reason is that the connecting cilia are fragile to the shearing force in the process of preparing TEM samples and OSs were broken off from organoids. Furthermore, previous studies reported that RPE cells are essential for photoreceptor differentiation and maturation^38^. It was observed that ROs would form better-stratified structures and more OS-like structures in the presence of RPE when some CiPSC-ROs obtained a small quantity of RPE cells (Fig. 2H). Thus, coculture of RPE with ROs seems to be necessary for outer segment morphogenesis as well as photoreceptor maturation.

The *Crx* gene plays a key role in photoreceptor development. In the current study, *Crx*-tdTomato reporter line CiPSCs were used to track the development of photoreceptors in vitro. The number of tdTomato^+^ cells and the tdTomato intensity continuously increased as the differentiation proceeded, and the proportion of tdTomato^+^ cells reached 85% at D32, which was consistent with the mature mouse retina in vivo (around 80%)^20^. Importantly, the tdTomato reporter could be a useful tool for transplantation application, as *Crx*-tdTomato^+^ photoreceptors could be detected and purified by fluorescence-activated cell sorting (FACS) without additional steps for labeling. In this study, *Crx*-tdTomato^+^ was also used to trace the transplanted photoreceptor cells in *rd* mice, we detected that tdTomato^+^ photoreceptors survived and established functional synaptic connections in the host retinae.

The cell replacement therapies using PSC-derived photoreceptors is a promising strategy to replace lost retinal cells and improve vision, which has been applied in various preclinical studies and clinical trials. In this study, small-molecule compounds were used to reprogram MEFs into CiPSCs, which were then differentiated into polarized and stratified 3D ROs containing various retinal cell types. Moreover, transplantation of CiPSC-PR could significantly improve visual function, and transplanted photoreceptors could survive, establish functional synaptic connections in the recipient retina. In this study, analysis of retinal function was performed using PLR test and functional recovery was detected in eyes with CiPS-PR transplants compared with the control animals. Meanwhile, we also conducted full-field ERG measurements on *rd10* mice four weeks after transplantation (data not shown). Due to the severe atrophy of *rd10* mice retina at the time of detection, ERG itself is not sensitive to show functional improvements between the transplant group and the control group. Most of the previously published studies on photoreceptors transplantation did not show functional recovery in full-field ERG test, and PLR test has always been the main experiment for evaluating the functional repair of photoreceptors transplantation^26,27^. Therefore, we thought that the results of PLR test could reflect the restoration of visual function after transplantation in this research.

In summary, this study established small compound reprogramming approaches to inducing fibroblasts into functional retinal cells, avoided the ethical issues and potential risks of genomic integration of exogenous genes by gene overexpression approaches, and opened up avenues for future applications of retinal organoids in clinical transplantation.

## Methods

### Mouse lines

The *Oct4*-GFP transgenic mouse (CBA/CaJ×C57BL/6J) was from the Jackson Laboratory. The C57BL/6 and *rd10* mice were from Shanghai Vital River Laboratory. All mice were housed at stable conditions (21 °C ± 2 °C) with a 12-h dark/light cycle. In strict adherence to ethical and legal requirements, we employ humane methods to terminate the lives of laboratory mice with the aim of minimizing or eradicating their suffering. Initially, we administer pentobarbital as an anesthetic agent and subsequently proceed with euthanasia through cervical dislocation once successful anesthesia is achieved. All animal experiments were approved by the Animal Ethics Committee of Beijing Tongren Hospital, China.

### Cell isolation and culture

The MEFs were isolated from E13.5 embryos as previously described^21^. Briefly, the head, limbs, visceral tissues, gonads, vertebral column, rib and sternum were removed from embryos. The remaining parts were washed twice with PBS (containing 2% penicillin-streptomycin (PS), Gibco), sliced into small pieces, dissociated with 0.25% trypsin-EDTA (Gibco) (3 ml per embryo) at 37 °C for 5–10 min, and plated into 10 cm dishes in MEFs culture medium containing DMEM basic (GIBCO), 10% fetal bovine serum (FBS, Gibco), 1% GlutaMAX (Gibco), 1% nonessential amino acids (NEAA, SIGMA), 0.1 mM 2-mercaptoethanol (Sigma) and 1% penicillin-streptomycin (PS, Gibco). In this study, we used MEFs within three passages to avoid replicative senescence.

The CiPSCs were maintained on a feeder layer of mitomycin C-treated MEFs in ESCs culture medium containing GMEM (Gibco), 10% knockout serum replacement (KSR, Gibco), 10% FBS, 1% GlutaMAX, 1% NEAA, 0.1 mM 2-mercaptoethanol, and 2iL (3 mM CHIR99021, 1 mM PD0325901 and 1000 U/mL LIF). The 2iL should be freshly added to the culture medium each time at medium change. The CiPSCs were passaged every 3 days. The MEFs and CiPSCs were cultivated at 37 °C with 5% CO_2_.

### Small-molecule compounds and libraries

The small molecules: VPA (V), CHIR99021 (C), 616452 (6), Tranylcypromine (T), Forskolin (F), AM580 (A), DZNep (Z), 5-aza-dC (D) were from Sigma. The EPZ004777 (E) and SGC0946 (S) were from Selleck. The bFGF was from BD. All chemical components were described in the Supplementary Table S1.

### Reprogramming medium preparation

The VC6TF chemical medium contains KnockOut DMEM (Gibco), 10% KSR (Gibco), 10% FBS (Gibco), 1% GlutaMAX (Gibco), 1% NEAA (Gibco), 0.055 mM 2-mercaptoethanol (Sigma), 1% PS (Gibco), and 100 ng/ml bFGF (BD). On D0-12, small molecules were added into the medium: VPA (0.5 mM), CHIR99021 (20 μM), 616452 (10 μM), Tranylcypromine (5 μM), Forskolin (50 μM), AM580 (0.05 µM), EPZ004777 (5 µM). On D12-16, the concentrations of bFGF, CHIR99021 and Forskolin were reduced to 25 ng/ml, 10 μM and 10 μM, respectively. The medium should be shaken for at least 30 min to ensure that all components are fully dissolved.

The VC6TFAZDS chemical medium contains KnockOut DMEM (Gibco), 10% KSR (Gibco), 10% FBS (Gibco), 1% GlutaMAX (Gibco), 1% NEAA (Gibco), 0.055 mM 2-mercaptoethanol (Sigma), 1% penicillin-streptomycin (Gibco), 25 ng/ml bFGF (BD) and supplemented with small molecules of 0.5 mM VPA, 10 μM CHIR99021, 10 μM 616452, 5 μM Tranylcypromine, 10 μM Forskolin, 0.05 µM AM580, 0.05 μM DZNep, 0.5 μM 5-aza-dC, and 5 μM SGC0946. The medium should be shaken for at least 30 min to ensure that all components are fully dissolved.

The N2B27-2iL medium contains 47% DMEM/F12 (Gibco), 47% Neurobasal (Gibco), 1% N2 supplement (Gibco), 2% B27 supplement (Gibco), 1% GlutaMAX (Gibco), 1% NEAA (Gibco), 1% penicillin-streptomycin (Gibco), 0.055 mM 2-mercaptoethanol (Sigma), and 2iL (3 mM CHIR99021, 1 mM PD0325901 and 1000 U/mL LIF). The 2iL should be freshly added to the culture medium each time at medium change. The medium should be shaken for at least 30 min to ensure that all components are fully dissolved.

### CiPSCs induction^13^

In brief, MEFs were seeded at a density of 5 × 10^4^ cells per well of a 6-well plate with MEFs culture medium. The plate was pre-coated with 0.1% gelatin for 30 min and washed by DPBS (Gibco) three times. After overnight culture, MEFs were first cultured in VC6TF chemical medium for 16 days, and then, small molecules DZNep, 5-aza-dC and SGC0946 were added into the reprogramming medium (VC6TFAZDS chemical medium) for the next 12 days. On D28, the medium was replaced to N2B27-2iL medium for the last 12 days. On D40, *Oct4*-EGFP^+^ colonies were picked up and seeded on feeder cells with ESCs culture medium for further expansion (P1).

### Differentiation of CiPSCs into 3D retinal organoids

The CiPSCs were differentiated into 3D ROs using the SFEBq protocol as previously described^15^. At D0, CiPSCs were dissociated into single cells. After feeder cells were removed, CiPSCs were plated in a 96-well low adhesion plate (Corning) at a density of 3–5 × 10^3^ cells per well in 100 μl retinal differentiation medium that contains GMEM (Gibco), 1.5% KSR (Gibco), 1% NEAA (Gibco), 1% sodium pyruvate (Sigma) and 0.055 mM 2-mercaptoethanol (Sigma). The next day (D1), a volume of 240 µl Matrigel (>9.5 mg/ml) (Corning) was diluted with 1.8 ml of retinal differentiation medium. An aliquot of 20 μl diluted Matrigel was added into each well to a final concentration of 2% (vol/vol). At D7, the aggregates were transferred into a 10 cm bacterial-grade petri dish with 10 ml retinal maturation medium I that contains DMEM/F12 with GlutaMAX (Gibco), 1% N2 supplement (Gibco), 0.055 mM 2-mercaptoethanol (Sigma) and 1% penicillin-streptomycin (Gibco). At D10, the organoids were cut into three evenly-sized pieces (trisection) to promote better organoid growth and maturation in the following days^39^. Then, the isolated retinal pieces were transferred to bacterial-grade petri dishes with 10 ml of retinal maturation medium II that contains DMEM/F12 with GlutaMAX (Gibco), 10% FBS, 1% N2 supplement (Gibco), 0.055 mM 2-mercaptoethanol (Sigma), 1% penicillin-streptomycin (Gibco), 1 mM taurine (Sigma) and 0.5 µM retinoic acid (RA, Sigma). At D14, the medium was refreshed with retinal maturation medium II without 0.5 µM RA. From D14 and onwards, half medium changes were carried out every 3 days with retinal maturation medium II without 0.5 µM RA. The cultures were incubated in 5% O_2_ from D0 to D10 and in 20% O_2_ from D10 onwards^15,30^.

### In vitro differentiation and in vivo teratoma formation

For in vitro differentiation, CiPSCs were cultured in a low adhesion dish with differentiation medium that contains GMEM, 10% FBS, 1% GlutaMAX, 1% NEAA and 0.055 mM 2-mercaptoethanol. After 3 days, the embryoid bodies were formed and transferred into adhesive dishes pre-coated with 1% gelatin, and cultured for 7 days. The cells in aggregates were then analyzed for expression of cell markers of three germ lineages with immunofluorescence staining. For teratoma formation in vivo, a total of 1 × 10^6^ CiPSCs were injected into NOD-SCID mouse and teratoma was formed 4 weeks later (*n* = 3). The control mice were injected with the same amount of MEFs and did not find the formation of teratoma (*n* = 3). Then teratomas were embedded in paraffin and processed with hematoxylin and eosin staining.

### Immunohistochemistry

For immunofluorescence staining, cells were cultured on glass coverslips in advance, fixed in 4% PFA (Byotime) for 15 min, and washed twice with DPBS. For immunofluorescence staining of organoids, aggregates were fixed in 4% PFA for 0.5 h, washed twice with DPBS, and embedded in OCT (Thermo-Fisher) and stored at −80 °C. For immunofluorescence staining of mouse retina, the mouse eyes were fixed in 4% PFA for 1 h, washed twice with DPBS, and cryoprotected in 15% (vol/vol) sucrose for at least 2 h and 30% (vol/vol) sucrose overnight at 4 °C, then they were embedded in OCT (Thermo-Fisher) and stored at −80 °C. These embedded organoids and mouse eyes were sectioned at 10 µm thickness with a Cryostat (Thermo-Fisher) before staining. Cells or sections were then blocked in 0.5% Triton X-100 and 4% bovine serum albumin (BSA) for 30 min at room temperature, incubated with primary antibodies diluted in 0.5% Triton X-100 and 1% BSA at 4 °C overnight, and then washed twice with DPBS. The secondary antibodies were diluted in 0.5% Triton X-100 and 1% BSA, and the incubation was lasted for 1 h at room temperature. Cell nuclei were stained with DAPI for 5 min, followed by washing three times with DPBS. Images were captured with a confocal microscopy (Leica). For comparison of intensity, images were captured using identical settings without overexposure. Antibodies used in this study were listed in the Supplementary Table S2.

### Quantitative reverse transcription PCR (qRT-PCR) and RNA sequencing

Total RNA was extracted from ROs and mouse retinae using the RNeasy Mini Kit (Qiagen) and the TRIzol reagent (Life Technologies) according to the manufacturer’s protocols. The mRNA was converted into cDNA with the iScript cDNA synthesis kit (Bio-Rad). The PCR was carried out following the protocol of the FAST SYBR Green Master Mix (Roche). All PCRs were performed in triplicate, and the expression of individual genes was normalized to that of *Gapdh*. The primers used for qRT-PCR were listed in Supplementary Table S3.

For RNA-seq, a total of 12 µg of RNA from ROs of a series of differentiation times (D14, 21, 28 and 35) was used for Illumina library preparation (Biomarker Technologies, Beijing, China). Statistical significance was determined by an adjusted *p* value < 0.05.

### Flow cytometry analysis

To prepare single-cell suspensions of *Crx*-tdTomato organoids, aggregates were dissociated with 0.25% trypsin-EDTA (Gibco) supplemented with 10 mg/ml DNase (Sigma) at 37 °C for 5–8 min, and cells were resuspended in DPBS containing 10% FBS and 10 mg/ml DNase. Cells were filtrated through 40 μm cell strainers before analysis. FACS analyses were performed with a FACS Calibur instrument (BD Biosciences). The results of tdTomato^+^ incidence at different timings were from at least three different organoids.

### Transmission electron microscopy (TEM)

For transmission electron microscopy imaging, ROs were fixed in 2.5% glutaraldehyde and 2% formaldehyde in 0.1 M cacodylate buffer (pH 7.2) and then processed for ultrathin sectioning (RMC-PXL). The images were taken with the electron microscope (H7500) equipped with a digital camera.

### Preparation and subretinal transplantation of CiPSC-derived photoreceptors

The D18 ROs were digested using Accutase (Stem Cell) for 60 min at 37 °C, then filtered to remove residual aggregates. After cell counting, the cells were centrifuged and resuspended in the medium at a concentration of 500,000 cells/μl.

The CiPSC-photoreceptor transplantation was performed on *rd10* mice. All mice were treated with general anesthesia using 1% pentobarbital sodium. The mouse pupils were dilated with 0.5% tropicamide for 10 min. Eyes were kept moist with cornea gel drops. Under an operating microscope, a small incision was created away from the lens but near the sclera with a sharp 33 G hypodermic needle (Hamilton, Germany). One microliter of cell suspension containing 500,000 cells was injected into the subretinal space between the retina and retinal pigment epithelium (RPE), resulting in a small bullous retinal detachment. The same volume of DPBS was injected into the other eye as experiment control. The mice were placed in the greenhouse after surgery until they woke up.

### Pupillometry

According to a previously published protocol^26^, the head and body of a mouse were put in a special plastic box in advance to keep the head unmoved under anesthesia. Then the mice were subjected to dark adaptation for at least 12 h. Using pupilometer (A2000; Neuroptics Inc., USA) combined with Bandicam software (Bandicam Company, South Korea), pupil images under different intensities of white light stimulation were recorded, and the ImageJ software was used to further analyze the pupil areas before and after light stimulation. The percentage of pupil contraction was calculated by comparing the pupil area before and after light stimulation at different intensities.

### Statistical analysis

The statistical analysis of PLR results was performed using ANOVA with Tukey post hoc tests, while the other data was analyzed by unpaired Student’s *t* test, t’ test or Dunnett T3 test. All statistical analyses were performed using GraphPad Prism (version 9.0; GraphPad Software, San Diego, CA). The *P* values < 0.05 were considered statistically significant.

## Supplementary information

Supplementary Information

## Data Availability

All data are available in the Article and its Supplementary Information. Raw of RNA-seq have been deposited at the NCBI GEO under accession numbers GEO: GSE137128 (mouse retinae), and Genome Sequence Archive (GSA): CRA020170.
