# Supplementary material for: Transplantation of derivative retinal organoids from chemically induced pluripotent stem cells restored visual function
Source: NPJ Regen Med. 2024 Dec 27;9:42. doi: 10.1038/s41536-024-00387-7 (PMC11681058; doi:10.1038/s41536-024-00387-7)
Supplement: Supplementary file 1 — Supplementary Information [file 41536_2024_387_MOESM1_ESM.pdf]

Supplemental Information

Transplantation of Derivative Retinal Organoids from Chemically Induced Pluripotent Stem Cells Restored Visual Function

Ning Zhao[1,#], Chang-Jun Zhang[1,#], Xiao Zhang[1], Wen Wang[1], Kangxin Jin[1], Zi-Bing Jin[1*]

1. Beijing Institute of Ophthalmology, Beijing Tongren Eye Center, Beijing Tongren Hospital, Capital Medical University, Beijing 100730, China.

[#]These authors contributed equally.

*Correspondence: Zi-Bing Jin, Beijing Institute of Ophthalmology, Beijing Tongren Eye Center, Beijing Tongren Hospital, Capital Medical University, Beijing 100730, China. E-mail: jinzibing@foxmail.com

Supplementary figure and table

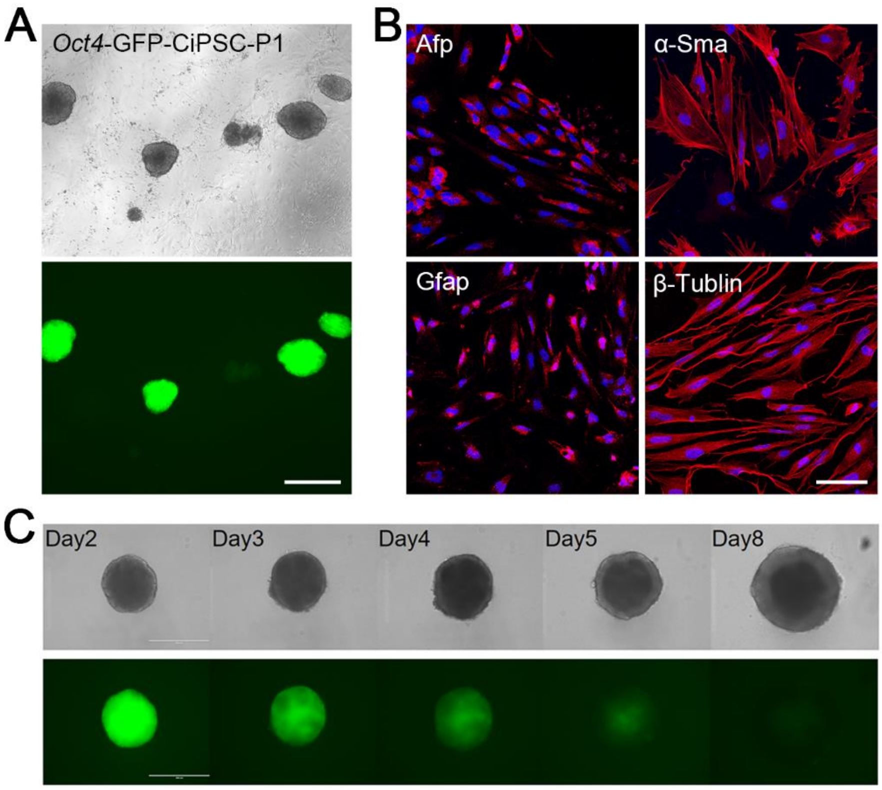

Supplementary figure 1. Characterization of CiPSCs (Related to Figure 1 and Figure 2)

(A) Representative bright field and fluorescence images from the primary CiPSCs (passage 1). Scale bar, 200 µm. **(B)** In vitro differentiation of CiPSCs. Immunostaining of cell markers from endoderm (Afp), mesoderm (a-Sma) and ectoderm (Gfap, β-Tublin) is shown. Scale bar, 100 µm. **(C)** Morphological and fluorescence changes at distinct timepoints during differentiation using *Oct4*-GFP reporter CiPSCs. Images were captured using the same settings. Scale bar, 100 µm.

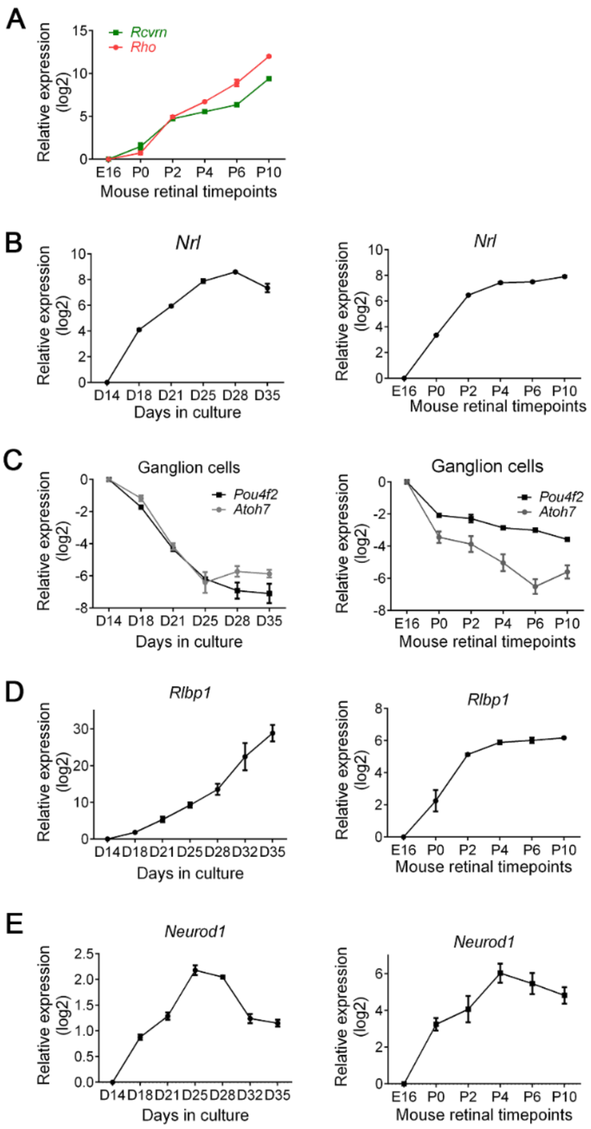

Supplementary figure 2. Expression of cell-specific markers of photoreceptors, ganglion cells, Müller glia cells and interneurons in retinal organoids and mouse retina (Related to Figure 3 and Figure 4)

(A, B) The qRT-PCR analysis of photoreceptor cell-relevant genes (*Rcvrn*, *Rho*, *Nrl*) in ROs and/or mouse retinae. Average expression values for each gene were further normalized to that of E16 to yield relative expression values (log2 scale). Data was represented as means ± SEM. **(C-E)** The qRT-PCR analysis of cell-specific gene expression in retinal ganglion cells (*Pou4f2*, *Atoh7*), Müller glia cells (*Rlbp1*), interneurons (*Neurod1*)-relevant gene expression in ROs (left) and mouse retinae (right). Average expression values for each gene were further normalized to that of E16 to yield relative expression values (log2 scale). Data was represented as means ± SEM.

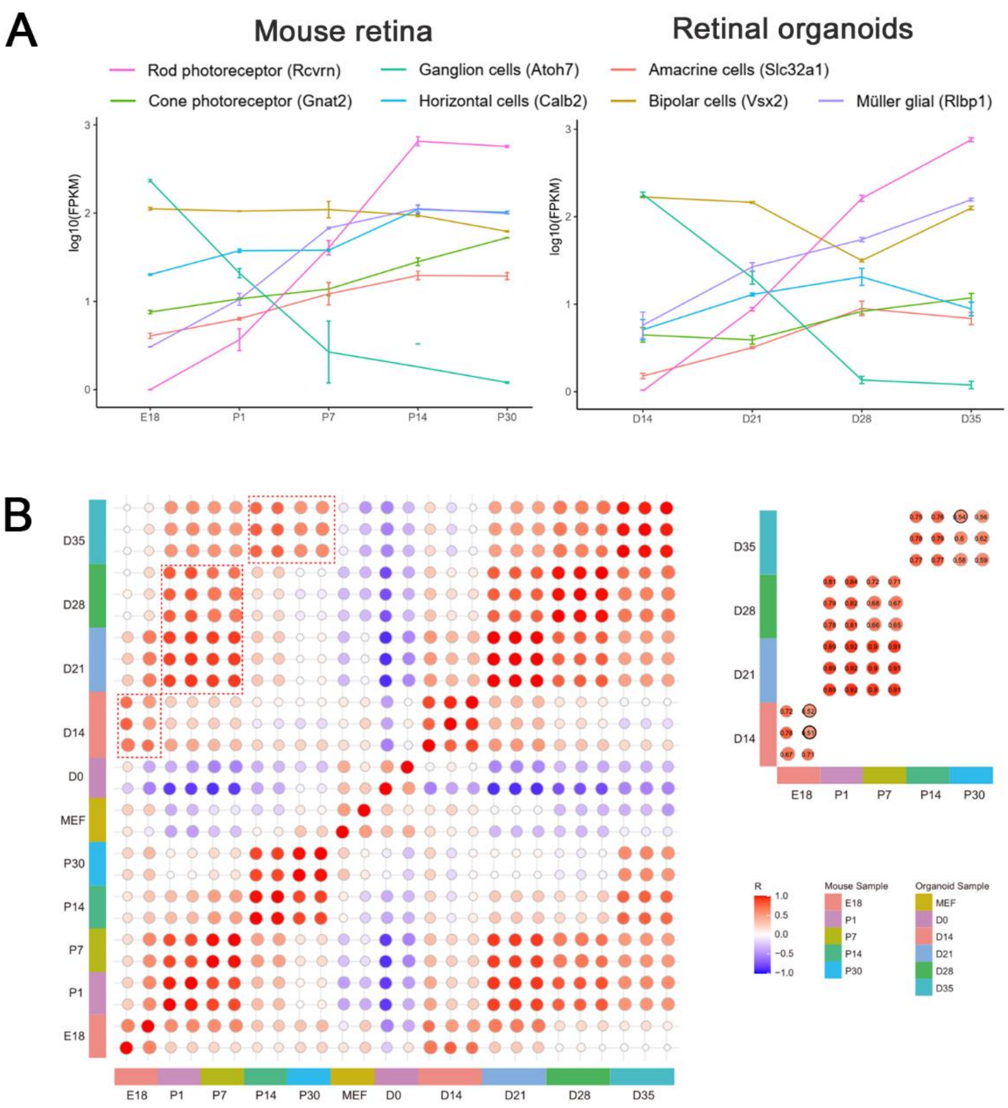

Supplementary figure 3. Dynamic transcriptome analysis of developing retinal organoids (Related to Figure 5)

(A) Heatmaps showed the expression of selected cell type-specific genes in ROs and *in vivo* mouse retinae. The average expression value (counts per million [cpm]) at each timepoint is plotted in log2 scale. The color scale bars refer to gene expression. Three biological sample replicates were used at each timepoint. **(B)** Pearson correlation plots calculated using the expressed gene CPM values of selected retina-related genes from the ROs and *in vivo* mouse retinae dataset. Each dot represents an independent sample. R = Pearson correlation coefficient. The numbers inside the circles represent the R value.

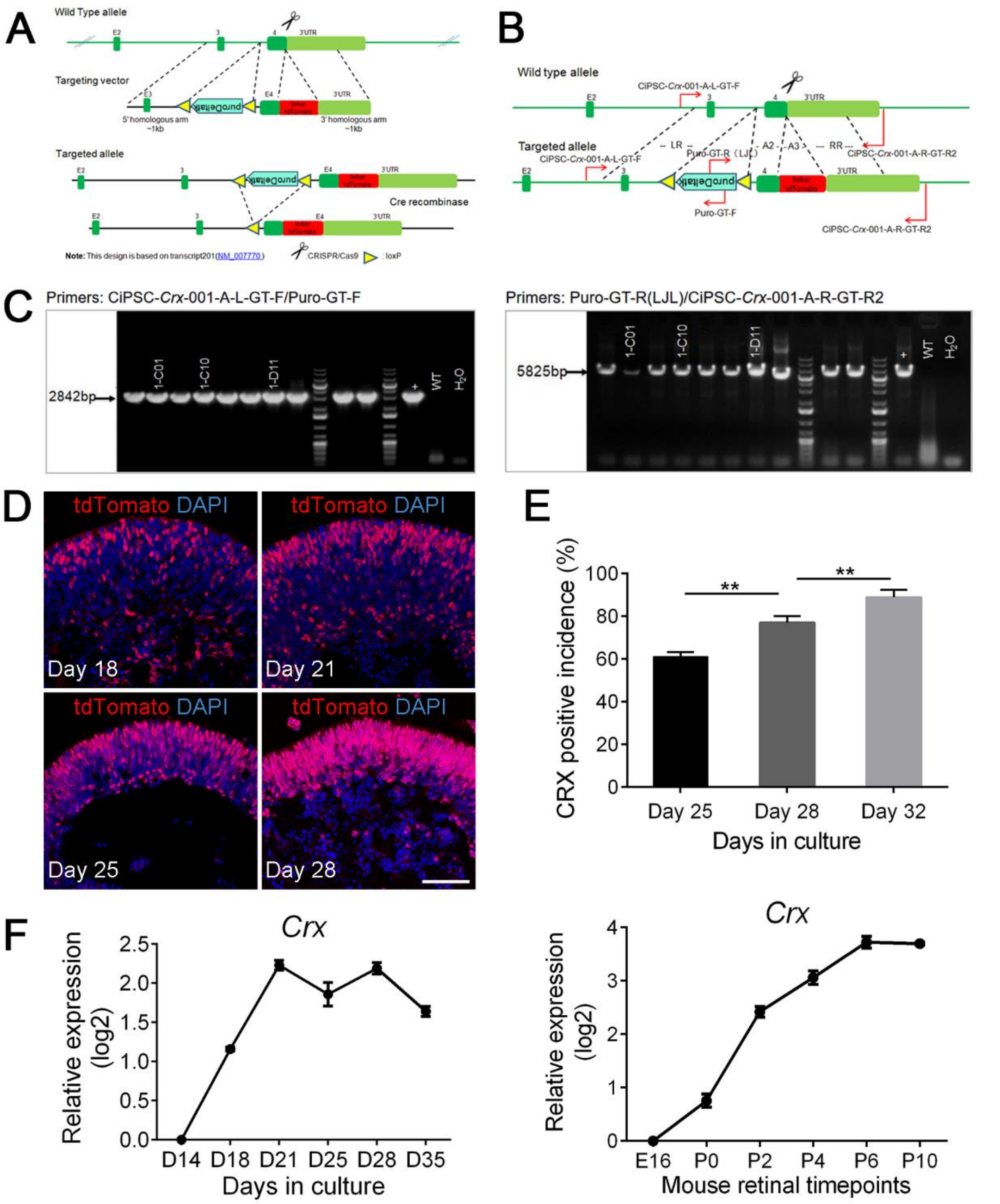

Supplementary figure 4. Generation of *Crx*-tdTomato reporter photoreceptors from CiPSCs (Related to Figure 6)

(A) Schematic of the construction of the *Crx*-tdTomato reporter line using CRISPR-Cas9. **(B)** Schematic of the primer designed for *Crx*-tdTomato labeled CiPSC clone identification. **(C)** The *Crx*-tdTomato reporter CiPSCs were identified and isolated through amplification of single-cell clones and screening of the PCR products. **(D)** The expression of tdTomato (red) in ROs at distinct timepoints during differentiation. Images were captured using the same confocal settings. Scale bar, 50 μm. **(E)**

Quantitative analysis of tdTomato^{+} cells by flow cytometry analysis. The control tdTomato^{-} cells were based on CiPSCs-derived ROs without tdTomato at the same differentiation day. Data was represented as means ± SEM. N > 3, ** $p < 0.01$. **(F)** The qRT-PCR analysis of *Crx* in ROs and mouse retinae. Average expression values for *Crx* in mouse retina were further normalized to that of E16 to yield relative expression values (log2 scale). Data was represented as means ± SEM.

A

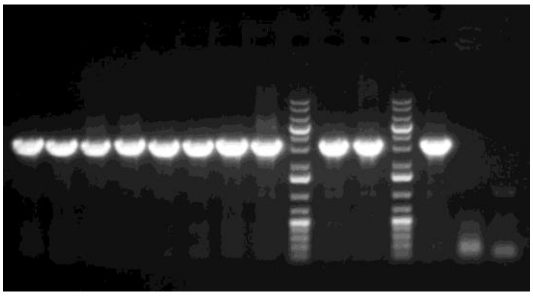

B

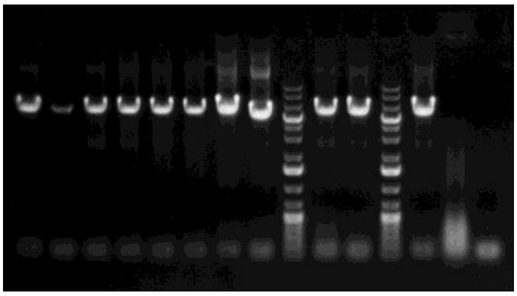

Supplementary figure 5. The uncropped and unprocessed gel images of Supplementary figure 4C.

(A) The uncropped and unprocessed gel images of Primer: CiPSC-*Crx*-001-A-L-GT-F/Puro-GT-F. **(B)** The uncropped and unprocessed gel images of Primer: Puro-GT-R(LJL)/CiPSC-*Crx*-001-A-R-GT-R2.

Supplementary table 1: Information of the small molecule compounds in this study.

Chemical	Structure	M.W.	Com & Cot
Valproic acid sodium salt (VPA, V)	$(CH_3CH_2CH_2)_2CHCOO^-$ Na^+	166.19	Sigma P4543
CHIR99021 (C)	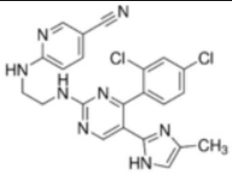	501.8	Sigma SML1046-25MG
RepSox (6)	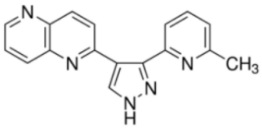	287.32	Sigma R0158-25MG
Tranylcypromine (T)	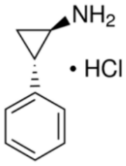	169.65	Sigma P8511
Forskolin (F)	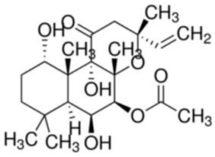	410.50	Sigma F6886
AM580 (A)	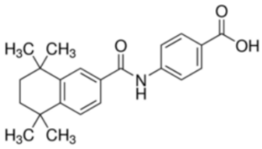	351.44	Sigma A8843
EPZ004777 (E)	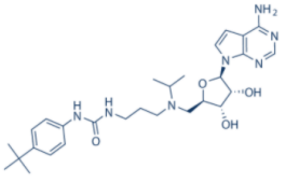	539.67	Selleckchem S7353
3-Deazaneplanocin A (DZNep, Z)	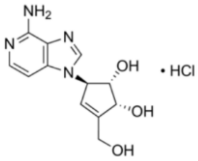	298.73	Sigma SML0305
5-aza-dC (D)	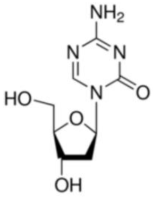	228.21	Sigma A3656
SGC0946 (S)	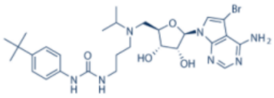	618.57	Selleckchem S7079

Retinoic acid (RA)	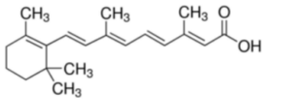	300.44	Sigma R2625-100MG
SU5402	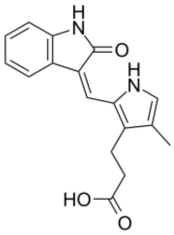	296.32	Sigma SML0443-5MG
Y27632	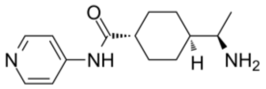	247.34	Selleck S1049
L-taurine	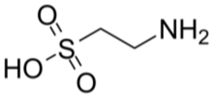	125.15	Sigma T8691-25G

Supplementary table 2: Antibody information and dilutions.

Antibody	Species	Company	Cat. No.	Dilution
Oct4 antibody	rabbit	Abcam	ab18976	1:250
Nanog antibody	rabbit	Abcam	ab109250	1:250
Sox2 antibody	mouse	Santa Cruze	sc-365823	1:100
AFP antibody	mouse	RD	MAB1368-SP	1:300
α-SMA antibody	mouse	Abcam	ab7817	1:250
GFAP antibody	mouse	Sigma	HPA056030	1:1000
β-TUBLIN antibody	mouse	Santa Cruze	sc-166729	1:100
Pax6 antibody	rabbit	Biolegend	B214847	1:300
Vsx2 antibody	rabbit	Sigma	AV31974	1:1000
Rax antibody	mouse	Santa Cruze	sc271889	1:100
Otx2 antibody	rabbit	Abcam	ab183951	1:250
Laminin antibody	mouse	Abcam	ab11575	1:250
ZO-1 antibody	rabbit	Invitrogen	SH255451	1:1000
PH3 antibody	rabbit	Abcam	ab80612	1:250
Ki67 antibody	mouse	BD	6280947	1:100
Pou4f1 antibody	mouse	Santa Cruze	sc-8429	1:100
Prox1 antibody	rabbit	Abcam	ab101851	1:250
Calretinin antibody	mouse	Santa Cruze	sc-365956	1:100
Rhodopsin antibody	mouse	Invitrogen	MA1-722	1:100

Recoverin antibody	rabbit	Abcam	ab5585	1:250
Rlbp1 antibody	mouse	Abcam	ab15051	1:200
Prkca antibody	rabbit	Invitrogen	PA5-17551	1:50
Arl13b antibody	rabbit	Proteintech	17711-1-AP	1:200
Synaptophysin antibody	mouse	Abcam	ab32127	1:200
Ctbp2 antibody	mouse	RD	612044	1:200

Supplementary table 3: Primers used for PCR reactions.

Gene	Forward (5' to 3')	Reverse (5' to 3')
Oct4	AGTGGGGCGGTTTTGAGTAA	TTCCAAAGAGAACGCCCAGG
Nanog	AGGACAGGTTTCAGAAGCAGA	CCATTGCTAGTCTTCAACCACTG
Sox2	GGAAAGGGTTCTTGCTGGGT	ACGAAAACGGTCTTGCCAGT
Lin28	CTTTGCCTCCGGACTTCTCT	GCGCACGTTGAACCACTTAC
Sall4	ATTACTGGGACATGCGCGTT	CAGTTCCCAGGGGAGTTCAC
Gapdh	CTAAGGCCAACCGTGAAAAG	ACCAGAGGCATACAGGGACA
Vsx2	CTCCGATTCCGAAGATGTTTCC	ATCTGGGTAGTGGGCTTCATT
Pax6	CTGGAGAAAGAGTTTGAGAGG	TGATAGGAATGTGACTAGGAG
Pou4f2	AGAAATCCCACCGCGAGAAG	TTGGCTGGATGGCGAAGTAG
Rcvrn	TACGACGTAGACGGCAATGG	TCCTCCTCTGTAAGTTTATCATCCT
Nrl	CCAAATCGCTACCTGTGGTT	GGGAACTCATCTCCAGCAAA
Rho	CCCTTCTCCAACGTCACAGG	TGAGGAAGTTGATGGGGAAGC
Rax	ATCCCAAGGAGCAAGGAGAG	TTCTGGAACCACACCTGGAC
Rlbp1	GGCACTTTCCGCATGGTTC	CCGGGTCTCCTCCTTTTCAT
Foxn4	CATGAAGGAGCACTTCCCCTA	TTTCCGGGCGGTCTGAGAT
Atoh7	ATCACCCCTACCTCCCTTTCC	CGAAGAGCCTCTGCCCATA
Neurod1	ACCTTTTAACAACAGGAAGTGGA	CTCATCTGTCCAGCTTGGGG
